# Stimulated scattering effects in gold-nanorod-water samples pumped by 532 nm laser pulses

**DOI:** 10.1038/srep11964

**Published:** 2015-07-15

**Authors:** Jiulin Shi, Haopeng Wu, Juan Liu, Shujing Li, Xingdao He

**Affiliations:** 1 Jiangxi Engineering Laboratory for Optoelectronics Testing Technology, Nanchang Hangkong University, Nanchang, 330063, China; 2 Key Laboratory of Nondestructive Test (Ministry of Education), Nanchang Hangkong University, Nanchang, 330063, China

## Abstract

Stimulated scattering in gold-nanorod-water samples has been investigated experimentally. The scattering centers are impurity particles rather than the atoms or molecules of conventional homogeneous scattering media. The pump source for exciting stimulated scattering is a pulsed and narrow linewidth second-harmonic Nd: YAG laser, with 532 nm wavelength, ~8 ns pulse duration, and 10 Hz repetition rate. Experimental results indicate that SMBS, SBS and STRS can be generated in gold-nanorod-water samples under appropriate pump and absorption conditions. The incident pump energy has to be larger than a certain threshold value before stimulated scattering can be detected. The absorption coefficient of samples at 532 nm wavelength depends on the one of characteristic absorption bands of gold nanorods located around 530 nm. A critical absorption coefficient 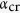 can be determined for the transition from SBS to STRS. Also, the spectral-line-broadening effects of STRS have been observed, the line-shape presents a pseudo-Voigt profile due to the random thermal motion of molecules and strong particle collision.

Stimulated light scattering in liquids is an important research topic in physics and chemistry^1^,^2^,^3^,^4^,^5^. Up to now, stimulated scattering effects have been reported usually in the following types: stimulated Brillouin scattering (SBS)^6^,^7^, stimulated Raman scattering (SRS) 8,9, and stimulated Rayleigh scattering (STRS)^10^,^11^. The nonlinear interaction between the optical field and acoustic phonons is responsible for SBS process: the pump wave generates acoustic wave through the process of electrostriction, and the acoustic wave scatters the pump wave through Bragg diffraction and produces the scattered light at Stokes frequency. This process can lead to an exponential growth of the Stokes energy if both the pump and the Stokes waves overlap over an extended propagation distance^12^. SRS is another remarkable phenomenon of nonlinear optical behavior that a large percentage of very high-power light may be transformed into molecular or atomic sidebands in the process of being transmitted through the materials^13^. STRS phenomenon was observed in linearly absorbing media and was attributed to light-induced localized thermal fluctuations^14^. In this process STRS presents an unusual feature that holds an anti-Stokes frequency shift of approximately one half of the half-intensity width of pump laser. The three well-known scattering mechanisms are always observed in a homogeneous scattering medium, for example, when intense coherent light is focused into a pure water sample, the backscattered light is known to consist of several components: stimulated Rayleigh scattering with the same frequency as the incident light, SBS with ~0.25 cm^−1^ frequency shift, and a SRS with ~10^4 ^cm^−1^ frequency shift^15^.

The development of nanotechnology has brought a favorable opportunity for researching the stimulated scattering effects in impurity particles. G. S. He *et al.* have reported a new type of stimulated scattering which is called stimulated Mie-Bragg scattering (SMBS)^16^. This newly stimulated scattering effect has been observed in a Mie-scattering medium that is gold nanorods of length comparable with incident light wavelength suspended in water, pumped by ~816 nm and ~10 ns laser pulses. The newly observed effect appears the features of no frequency shift and low pump threshold condition, and is different from most of other known stimulated scattering effects.

In this paper, we lay emphasis on the experimental observation of stimulated Brillouin and Rayleigh scattering in gold-nanorod-water samples pumped by a single longitudinal mode laser with ~532 nm wavelength, ~90 MHz line-width, and ~8 ns pulse duration. Experimental results indicate that the absorption coefficient of samples plays a crucial role during the stimulated scattering process. A critical absorption coefficient *α*_*cr*_ was determined for the transition from SBS to STRS.

**Samples and optical setup**

The samples for scattering measurements are cylindrical gold nanorods with ~13 nm diameter and ~90 nm length (the average aspect ratio is ~6.9) dispersed in water. Figure 1(a) shows the transmission-electron-microscope (TEM) image of cylindrical gold nanorods, and the transmission spectra of gold-nanorod-water samples with the different concentrations but with the same path length of 1 cm are shown in Fig. 1(b). The transmission spectra of samples were recorded by a SHIMADZU Solidspec-3700 UV-VIS-NIR scanning spectrophotometer. Comparing the transmission spectra of gold-nanorod-water samples with the transmission spectrum of pure water, we can see that, the transmission spectra of gold-nanorod-water samples possess two characteristic attenuation bands, one is located around 530 nm due to the transverse oscillation of electrons irrespective of the aspect ratio, and the other around 950 nm is due to the longitudinal oscillation of the electrons which depends on the aspect ratio of the gold nanorods.

Figure 2 shows the optical setup for measuring the stimulated scattering from the gold-nanorod-water samples. An injection-seeded, Q-switched and frequency-doubled Neodymium-doped Yttrium Aluminum Garnet (Nd: YAG) laser was used. The wavelength is ~532 nm, the pulse duration is ~8 ns, the repetition rate is 10 Hz, the divergence angle is ~0.45 mrad, and the beam size is ~12 mm. A narrow line-width (0.003 cm^−1^) can be achieved by switching on the seeder. The output energy of the laser was controlled by adjusting the time delay between the amplifier and the oscillator to keep the stability of the laser.

The polarization of the pump laser is horizontal, and becomes a circularly polarization along the 45^o^ direction after passing through the polarization beam splitter (PBS) and quarter-wave plate. A lens of 15 cm focal length was used to focus the pump laser beam into the sample cell. The stimulated scattering signals will be excited when the intensity of the pump laser is higher than a certain threshold level. The backscattered light changes the polarization from 45^o^ direction to vertical after passing through the quarter-wave plate, and was reflected by PBS. In this paper, PBS was used to split the backward scattering from the pump laser, it can reduce the noise influence to a certain extent. The spectral composition of the backscattered light was analyzed and photographed with the aid of a Fabry-Perot etalon (0.65 cm^−1^) and an intensified CCD (ICCD) camera (PI-MAX2 1003, Princeton Instrument). In order to reduce the reflection influence from the two optical surfaces of the sample cell, the normal direction of the front and the rear surfaces of the sample cell was slightly deviated from the direction of the pump laser beam with an angle of ~5°.

## Results And Discussions

**Figure 1:**
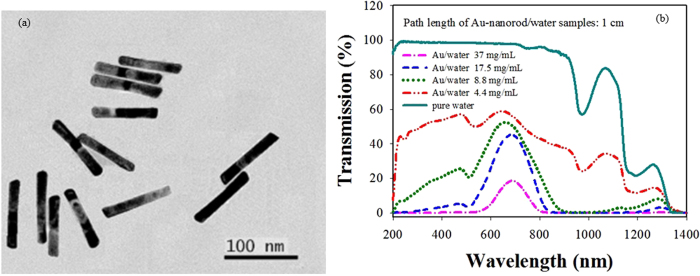
TEM image and transmission spectra of gold nanorods. (**a**) TEM image of gold nanorods used in stimulated scattering experiment. (**b**) Transmission spectra of 1 cm length gold-nanorod-water samples with different concentrations.

**Figure 3:**
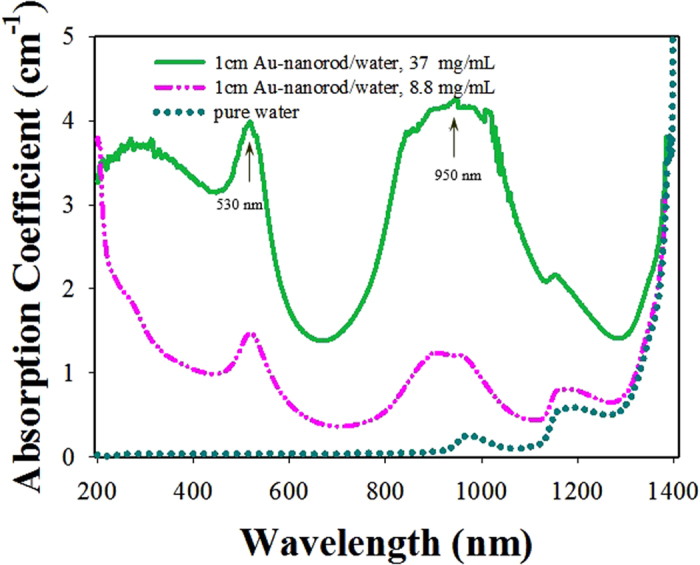
Measured absorption spectra of gold-nanorod-water samples by using spectrophotometer.

Figure 3 shows the measured absorption spectra of the gold-nanorod-water samples with two concentrations of 8.8 mg/mL and 37 mg/mL in the same path length of 1 cm. Corresponding to Fig. 1(b), the gold-nanorod-water samples hold two characteristic absorption bands that are located around 530 nm and 950 nm, respectively. Herman and Gray have reported theoretically that intense laser pulses travel through absorbing media can produce STRS from localized thermal fluctuations^10^. Rapid thermalization of the molecules because of the strong surface plasmon resonance absorption (SPRA) of gold nanorods at the laser frequency and the subsequent generation of large density fluctuations, coherent and localized, lead to the enhancement of the thermal density fluctuations already present in the samples, leading to enhancement of Rayleigh scattering process. Moreover, according to the Mie-scattering theory (the size of particles is comparable in order of magnitude to the incident wavelength), the gold nanorods suspended in water may provide a condition for producing an initial spontaneous Mie-scattering (seed) signal. The initial spontaneous Mie-scattering (seed) signal is an essential asset for the generation of so-called stimulated Mie-Bragg scattering (SMBS)^16^.

**Figure 4:**
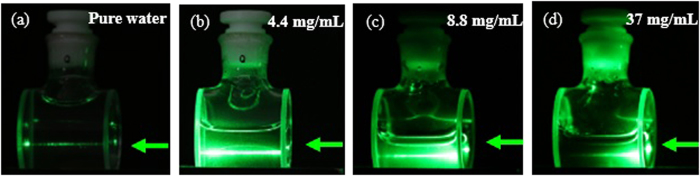
Scattering appearance in gold-nanorod-water samples with different concentrations pumped by 532 nm laser with the energy of 1.5 mJ/Pulse. The green arrows represent the propagation direction of incident pump laser.

**Figure 5:**
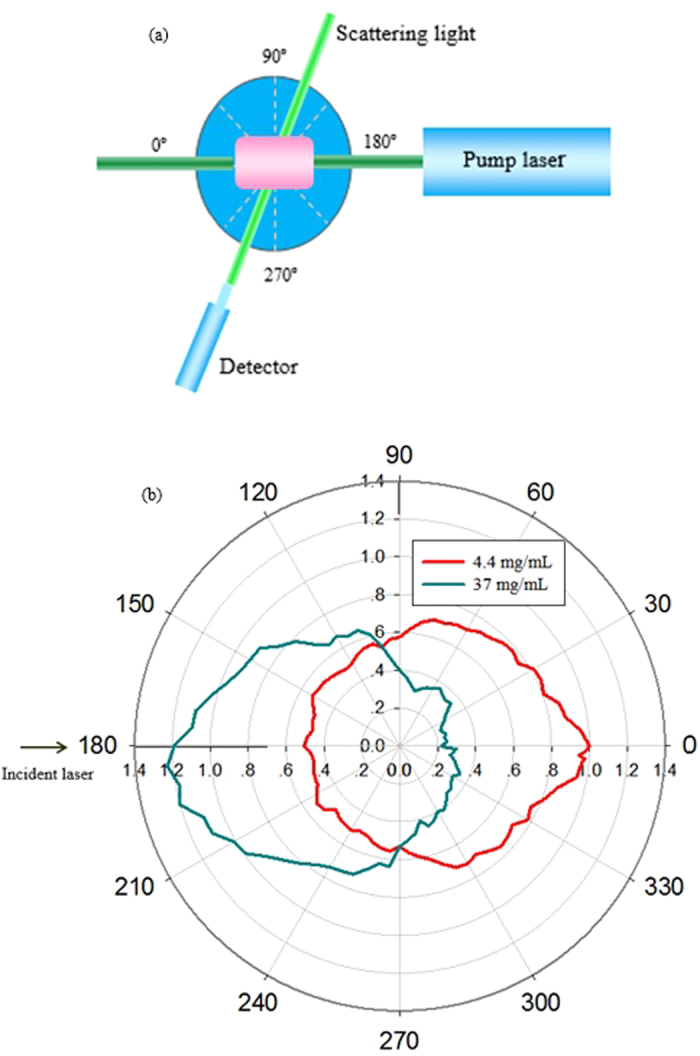
Experimental measurement. (**a**) Optical setup for measuring the scattering light of different angles. (**b**) Measured normalized angular dependence of scattering light from gold-nanorod-water samples.

Figure 4 shows the side view of gold-nanorod-water samples with different concentrations pumped by a weak-focusing 532 nm pulse laser with the energy of 1.5 mJ/Pulse. It can easily see that the scattering intensity and scattering position present a distinction from pure water to 37 mg/mL concentration. In pure water sample, as shown in Fig. 4(a), Rayleigh scattering can be observed from the side view. However, for gold-nanorod-water samples, there may be a Mie scattering in addition to the Rayleigh scattering, as shown in Fig. 4(b)–(d). In order to measure the angular distribution of the scattering light from the gold-nanorod-water samples, a setup geometry shown in Fig. 5(a) was employed. During measurements, the scattering light was collected by a lens, which was focused and fed to a photodiode detector. The scattering light was measured every 5 degrees over a range of 0^o^–360^o^. Shown in Fig. 5(b) is the measured normalized angular dependence of scattering intensity from gold-nanorod-water samples. It can be seen that, when the sample concentration increases from 4.4 mg/mL to 37 mg/mL, the intensity of scattering light was gradually weakening along with the propagation direction of pump laser.

**Figure 6:**
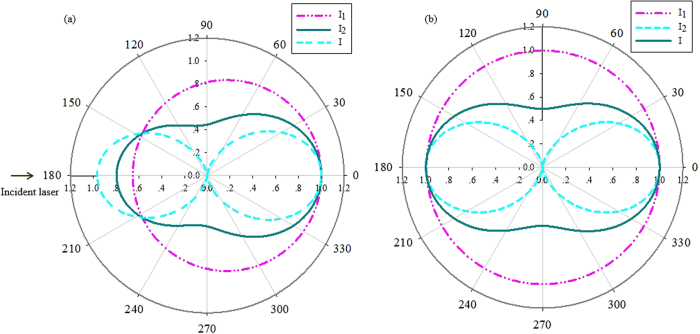
Calculated curves using the incident laser is 532 nm wavelength. (**a**) Based on the Mie-scattering theory. (**b**) Based on the Rayleigh-scattering theory.

As a comparison, Figure 6 presents the calculated normalized scattering curves in an angular coordinate based on Mie-scattering theory and Rayleigh-scattering theory, respectively. The wavelength of incident pump laser used in scattering theories was 532 nm, and the sizes of particles induced into calculating were 13 nm and 90 nm, respectively. In this figure, *I*_1_ (dash-dot-doted curves) corresponds to an incident laser with a linearly polarization along the direction perpendicular to the observation plane; *I*_2_ (dashed curves) corresponds to an incident laser with a linearly polarization along the direction parallel to the observation plane. In our experimental condition, the incident laser is a linearly polarization along the 45^o^ direction with the observation plane, so the intensity distribution (solid curves) can be calculated by using *I=I*_1_+*I*_2_/2 17. Comparing Fig. 5(b) and Fig. 6, it can be seen that the angular distribution of scattering intensity from the gold-nanorod-water samples is expressively different from the calculated results based on Mie scattering or Rayleigh scattering theory. There may be two possible reasons for this discrepancy: one is that the prediction of Mie or Rayleigh theory is based on an assumption of spherical scatterers, while the gold nanorods used in our experiments present cylindrical profile, it means that the scattering cross-section is different; the other is that stimulated scattering effects are excited in gold-nanorod-water samples when the incident pump laser energy is higher than a certain threshold value. Actually, we did observe two stimulated scattering effects that SBS and STRS in the backward direction. The experimental results together with analysis are presented as follows.

**Figure 7:**
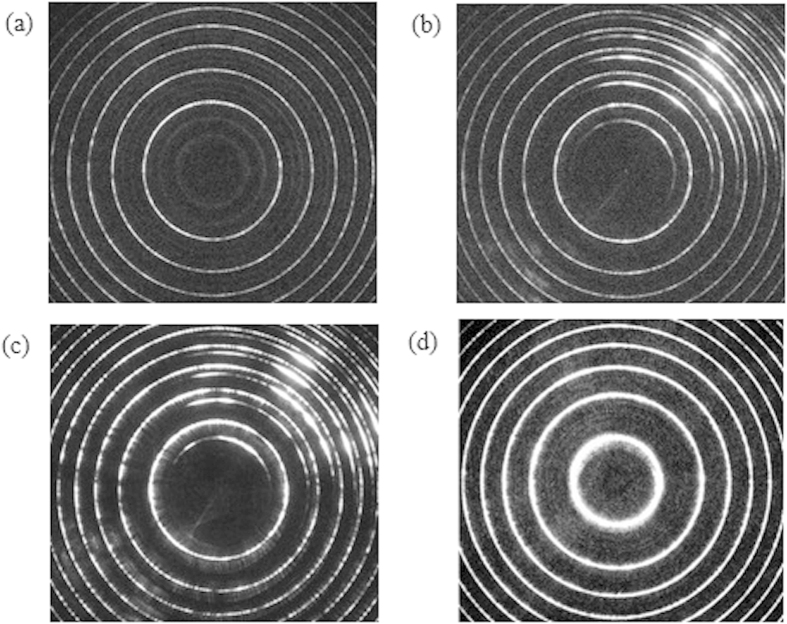
Measured Fabry-Perot interferograms of backscattered components in gold- nanorod-water samples. (**a**) and (**b**): spectra for pure water, the incident pump energy is ~0.43 mJ/Pulse and ~0.68 mJ/Pulse, respectively. (**c**) and (**d**): spectra for gold-nanorod-water samples with the concentration of 2.2 mg/mL, incident pump energy of ~3.15 mJ/Pulse and 3.4 mg/mL, incident pump energy of ~12.13 mJ/Pulse, respectively.

During the course of the experiment, it was found that when the incident pump energy exceeds a certain threshold value, a backward stimulated scattering light could be observed. Figure 7 shows the recorded Fabry-Perot interferograms of backscattered components in different sample concentrations. Shown in Fig. 7(a) is the interferogram formed by both the backward STRS (bright-rings) and spontaneous Brillouin scattering (dim-rings) in pure water, obtained by using the incident pump energy of 0.43 mJ/Pulse. It can be seen clearly that in the spectrum of spontaneous Brillouin scattering both Stokes and anti-Stokes components are observed. When the incident pump energy was increased further, SBS appeared in the neighborhood of the STRS line, extending toward the Stokes side, as shown in Fig. 7(b). There is no anti-Stokes component in the spectrum has been observed with certainty; for each interference order, there are two circles, the inner (half-rings) is the stimulated Brillouin component and the outer (whole rings) is the stimulated Rayleigh component. When the concentration of gold-nanorod-water sample was increased, as shown in Fig. 7(c), the intensity of Rayleigh component increases with the sample concentration and pump energy, but the Brillouin component will disappear when the concentration is increased beyond a certain value regardless of the pump energy.

**Figure 8:**
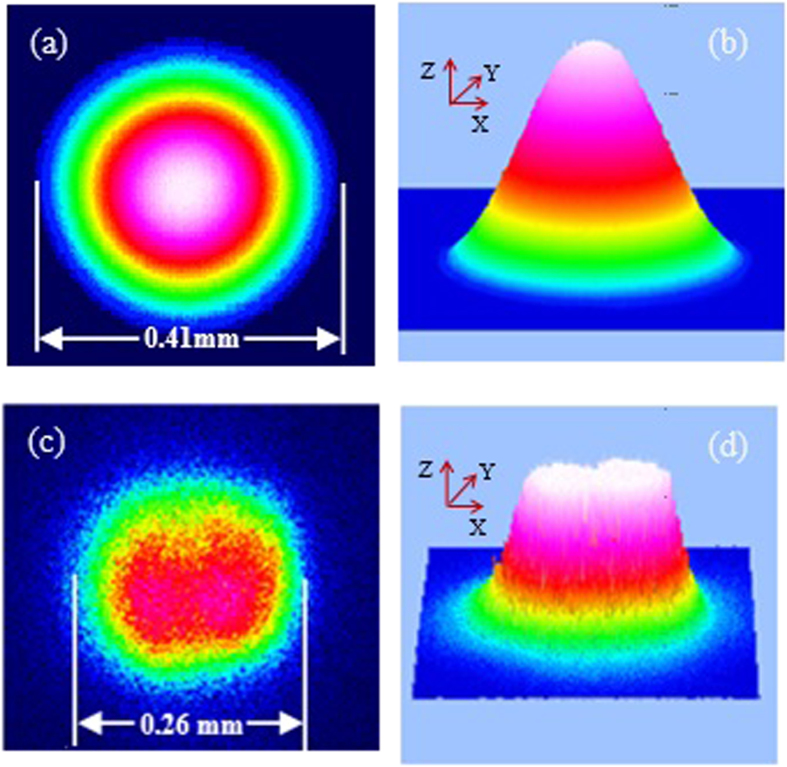
Measured far-field patterns of (**a**) transverse intensity distribution of the pump laser beam, (**b**) 3D Gaussian fit corresponds to (**a**), (**c**) transverse intensity distribution of the backward stimulated scattering beam, and (d) 3D Gaussian fit corresponds to (**c**). Sample concentration: 2.75 mg/mL. Pump energy: 3.15 mJ/Pulse.

Figure 8 shows the measured far-field patterns of the pump laser beam ((a), (b)) and backward stimulated scattering beam ((c), (d)). These images were obtained by focusing the pump laser beam and backward stimulated scattering beam via an *f *= 150 cm lens on the screen of CCD beam profiler (THORLABS BC106) located at the focal plane. From Figs. 8(a) we can see that the beam size for the backward stimulated scattering is smaller than the pump laser beam. Moreover, we found that the Gaussian-fit similarity of the transverse intensity distribution of pump laser is 81.95% in X direction and 82.38% in Y direction, respectively (Fig. 8(b)), but the transverse intensity distribution of the backward stimulated scattering presents a flat-top profile (Fig. 8(d)).

**Figure 9:**
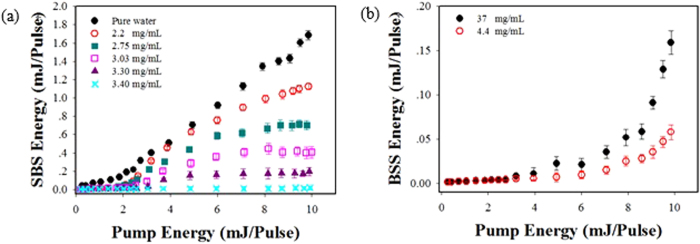
Change of SBS energy and STRS energy in different concentrations of gold-nanorod-water versus change of incident pump energy. (**a**) Energy relationship: incident pump laser vs. SBS; (**b**) Energy relationship: incident pump laser vs. STRS.

Figure 9 shows the changes of the energies of SBS and STRS with the change of the energy of the incident pump laser in different gold-nanorod-water sample’s concentrations. It can be seen from Fig. 9(a) that the SBS energy linearly increases with the increase of incident pump energy in pure water, but the slopes become lower and lower when the concentration of gold-nanorod-water samples was increased gradually. However, the STRS energy increases rapidly and exponentially when the incident pump energy was increased beyond a certain value, as shown in Fig. 9(b), at the same pump energy the sample with a higher concentration can produce a higher STRS output energy. After comparison between the results shown in Fig. 7 and Fig. 9, the following points could be obtained: the beginning of SBS or STRS in a gold-nanorod-water sample was observed to depend on two necessary conditions that the pump laser intensity *I*_p_ and the measured absorption coefficient *α* (or the sample concentration). First, the incident pump laser intensity has to be larger than the threshold intensity of SBS or STRS. Second, the absorption coefficient *α* should be made to outdo a critical value *α*_cr_ before STRS can be excited. The higher of the sample concentration is, the larger of the absorption coefficient of sample is.

It is necessary to state that, when pump energy is higher the multiple nonlinear effects can be induced due to high peak power. The others nonlinear effects will consume a part of energies of the pump laser, it will finally result in the reduction in the SBS and STRS energies. Therefore, the change of SBS energy and STRS energy presented in this paper is just a semi-quantitative description. In a real case, the multiple nonlinear effects, such as optical breakdown effect, self-focusing effect and stimulated Raman effect and so on, should be taken into account in a more rigorous stimulated process, which is beyond the scope of this work.

**Figure 10:**
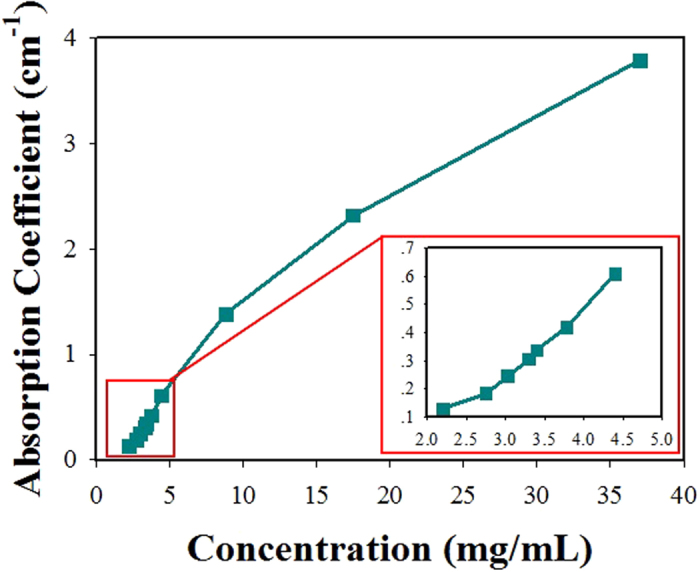
Measured absorption coefficients of gold-nanorod-water samples with different concentration values.

Figure 10 shows the measured absorption coefficients of gold-nanorod-water samples with different concentrations. The absorption coefficient *α* of the samples, determined from the measured absorbance at the wavelength 532 nm using spectrophotometer. As shown in Fig. 7, the measured absorption coefficients of the samples with the concentration of 2.2 mg/mL and 3.4 mg/mL, are 0.129 cm^−1^ and 0.336 cm^−1^, respectively. Both the Brillouin and Rayleigh scattering exist with different ratios of their intensities for the sample with the lower absorption coefficient of gold nanorods (Fig. 7(c)), but there is no Brillouin component and only the Rayleigh component appears when the absorption coefficient is beyond a certain critical absorption coefficient (Fig. 7(d)). In fact, the intensities of the Brillouin and Rayleigh components are approximately equal at an intermediate absorption coefficient by controlling the concentration value. Herman and Gray^10^ have reported that the absorption coefficient *α*_cr_ of an absorbing media for which the nonlinear gains for the Brillouin and Rayleigh scattering are equal is given by


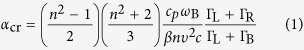


where 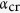 is the critical absorption coefficient, 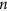 is the index of refraction of media, *c*_*p*_ is the specific heat at constant pressure, *β* is the volume expansion coefficient of media, *ω*_B_ is the frequency shift of the Brillouin scattering, 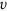 is the speed of sound in media, *c* is the speed of light in vacuum, and Γ_L_, Γ_R_ and Γ_B_ are the line-width of the pump laser, Rayleigh and Brillouin, respectively. If the absorption coefficient *α*<*α*_cr_, the SBS will be dominant during the backward stimulated scattering process. However, if the absorption coefficient *α*<*α*_cr_, SBS will be greatly suppressed, and the STRS will be dominant since the STRS draws most of the energy from the pump laser. In our experiment, we find that the estimated value of *α*_cr_ for the gold-nanorod-water samples is approximately 0.305 cm^−1^.

**Figure 11:**
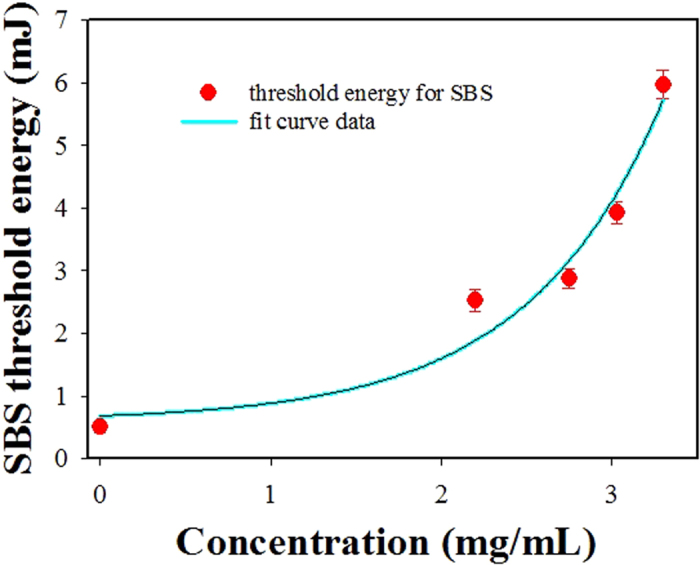
Measured pump threshold energies for producing SBS in Au-nanorod/water samples with different concentration values.

Moreover, the threshold energy for the appearance of the SBS was found to vary for gold-nanorod-water samples having different absorption coefficients. The measured pump threshold energies for generating SBS in different sample solution’s concentrations are shown in Table 1 and Fig. 11. We can see that the pump threshold energy for generating SBS becomes higher when the absorption coefficient (or concentration value) of the sample solution is increased. The pump threshold for the gold-nanorod-sample with the concentration of 3.3 mg/mL is about twelve times higher than the pure water sample, SBS will disappear when the absorption coefficient *α* is bigger than the critical absorption coefficient of *α*_cr _= 0.305 cm^−1^.

According to the results reported by Rank *et al.* and Cho *et al.*, the STRS of pulse light in absorbing media would experience an anti-Stokes shift due to a coupling between spontaneous thermal fluctuations and the incident pump laser^14^. They explained the anti-Stokes shift using a theory of one-photon-absorption-enhanced thermal density fluctuation, and the shift Δ*ω* being approximately equal to the predicted value given by


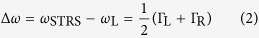


where, *ω*_STRS_ and *ω*_L_ are the angular frequencies of the stimulated thermal Rayleigh scattering and pump laser, respectively, Γ_L_ and Γ_R_ are the line-width of the pump laser and spontaneous thermal Rayleigh, respectively. Unfortunately, under our experimental conditions, there is no frequency shift can be observed between the backward stimulated thermal Rayleigh scattering and pump laser. It may, however, this disagreement be a result of superposition of the light due to the spectral-line-broadening in the backward stimulated scattering process. There may be two possible broadening mechanisms during the STRS process:

The first is the Doppler broadening due to the random thermal motion of molecules. As was known from the physical mechanism of the Doppler broadening, the line profile of the Doppler broadening presents a Gaussian profile. The full width at half maximum (FWHM) of the Gaussian profile is proportional to the thermodynamic temperature *T* of media:


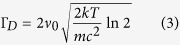


where, *v*_0_ is the center frequency of pump laser, *k* is the Boltzmann constant, *m* is the particle mass, *c* is the speed of light in vacuum, and *T* is the thermodynamic temperature of media. The intense laser pulses travel through absorbing gold-nanorod-water samples can produce rapid thermalization of the molecules because of the strong SPRA of gold nanorods at the laser frequency, leading to enhancement of Doppler broadening process.

The second possible broadening mechanism is that an extremely strong particle collision process can produce a Lorentzian profile. The FWHM of the Lorentzian profile is proportional to the thermodynamic temperature *T* and the concentration of particles *n*_*p*_:


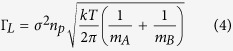


where, *σ* is the effective collision cross-section. The increase of the concentration of gold nanorods suspended in water boosts the Lorentzian broadening process.

**Figure 12:**
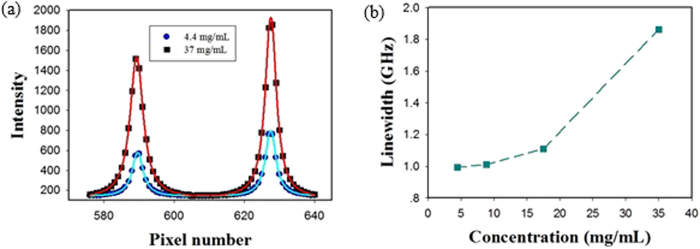
Measured linewidth of backward stimulated scattering in Au-nanorod/water samples with different concentration values. (**a**) Spectra recorded by F-P etalon and ICCD camera; (**b**) Linewidth at different concentration values.

To verify the above conclusion, as shown in Fig. 12, we did observe a broadening phenomenon during the backward stimulated scattering process. Figure 12(a) shows the measured spectra of the backward stimulated scattering through a 532 nm etalon and an ICCD camera. The fit curves used in figure are the pseudo-Voigt profile that resulting from the convolution of two broadening mechanisms: Gaussian profile (as a result of the Doppler broadening) and Lorentzian profile (as a result of the collision broadening). It can be seen clearly that, the line-width (FWHM) of spectrum of backward stimulated scattering in 37 mg/mL concentration is wider than that of 4.4 mg/mL concentration. The measured linewidth of backward stimulated scattering as a function of the concentration is shown in Fig. 12(b). From the measured data, we can also see that the sample solution with a higher concentration can produce a wider linewidth. Based on these experimental results, we conclude that the higher the concentration values of sample solution, the stronger the broadening effect.

## Conclusion

We have experimentally observed the backward stimulated scattering from gold-nanorod -water sample solution pumped by a 532 nm narrow-band pulse laser. The scattering centers are not the atoms or molecules of a conventional homogeneous scattering medium but impurity particles. Experimental results show that SMBS, SBS and STRS can be effectively generated in a gold-nanorod-water sample under appropriate pump and absorption conditions. First, the incident pump laser energy has to be larger than a certain threshold value before stimulated scattering can be detected. Second, SBS and STRS could simultaneously appear in the scattering process, but one of them dominated the other under a certain absorption coefficient *α*_cr_. Moreover, the spectral-line-broadening effects of STRS have been observed due to the random thermal motion of molecules and strong particle collision. The spectral-line-broadening effects are completely to the disadvantage of the observation of the anti-Stokes shift.

## Additional Information

**How to cite this article**: Shi, J. *et al.* Stimulated scattering effects in gold-nanorod-water samples pumped by 532 nm laser pulses. *Sci. Rep.* **5**, 11964; doi: 10.1038/srep11964 (2015).

## Figures and Tables

**Figure 2:**
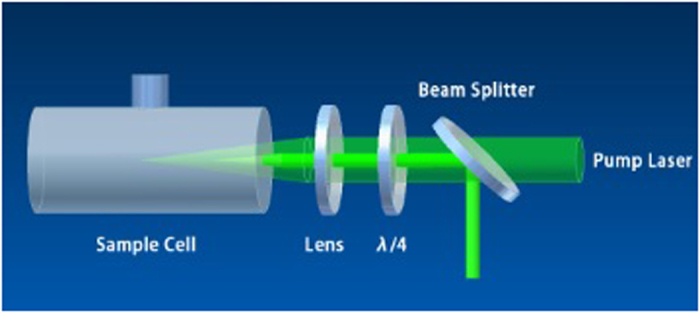
Setup geometry for backward stimulated scattering experiment. λ/4 indicates quarter-wave plate.

**Table 1:** Absorption coefficient and SBS threshold in different sample concentrations.

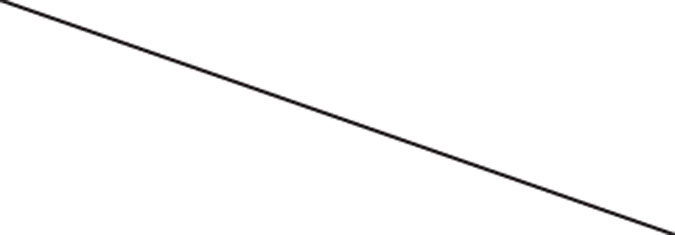	Sample concentration (mg/mL)										
	0	2.2	2.75	3.03	3.30	3.40	3.78	4.4	8.8	17.5	37
Absorption coefficient (cm^−1^)	0.041	0.129	0.183	0.247	0.305	0.336	0.417	0.606	1.38	2.316	3.833
SBS threshold energy (mJ)	0.51	2.53	2.88	3.92	5.97	—	—	—	—	—	—
Threshold ratio (to water)	1	4.96	5.65	7.69	11.7	—	—	—	—	—	—
